# Diagnosis and allergen immunotherapy treatment of polysensitised patients with respiratory allergy in Spain: an Allergists’ Consensus

**DOI:** 10.1186/2045-7022-4-36

**Published:** 2014-11-07

**Authors:** Carmen Vidal, Ernesto Enrique, Angeles Gonzalo, Carmen Moreno, Ana I Tabar, on behalf of Expert Clinical Participants

**Affiliations:** Allergy Departments of Complejo Hospitalario Universitario de Santiago, Rúa Ramón Baltar s/n, Santiago de Compostela, 15706 Spain; Allergy Departments of Hospital de Sagunto, Valencia, Spain; Allergy Departments of Hospital Universitario Infanta Cristina, Badajoz, Spain; Allergy Departments of Hospital Universitario Reina Sofía, Córdoba, Spain; Allergy Departments of Complejo Hospitalario de Navarra, Pamplona, Spain

**Keywords:** Consensus, Delphi method, Respiratory allergy, Polysensitisation, Diagnosis, Allergen immunotherapy

## Abstract

**Background:**

Polysensitisation is common in patients with respiratory allergy in Spain. Selection of the best allergen immunotherapy (AIT) is difficult in polysensitised patients. The present study was designed to help allergists better identify relevant allergens in these patients and to improve the selection of AIT in Spain.

**Methods:**

Sixty-two Spanish allergists answered a survey containing 88 items divided into four groups: 1) general approach to polysensitised subjects; 2) sensitisation profile involving mite, animal dander and moulds; 3) grass and olive pollen co-sensitisation, and 4) other pollen polysensitisation profile (weed and tree pollen). The Delphi method was used.

**Results:**

A consensus was achieved for 83% of items (92%, 81%, 83% and 73% of the four groups analysed, respectively). Only polysensitised patients with clinical relevance should be considered polyallergic. A detailed medical history (clinical symptoms and medication) together with a profound knowledge of allergens present in the patient’s environment are essential for diagnosis. Skin prick tests (SPTs) are not adequate to decide the clinical relevance of each allergen. Serum specific IgE against allergen sources adds value to SPT but molecular diagnosis, when possible, is strongly recommended, especially in pollen-allergic patients. Specific allergen challenge tests are difficult to perform and not recommended for daily practice. Regarding AIT composition, up to three allergens can be used in the same vaccine, but only related allergens may be mixed. In some cases more than one vaccine may be needed.

**Conclusion:**

Some criteria have been established to improve diagnosis and AIT prescription in polysensitised patients.

## Background

Respiratory allergy represents an overall public health problem because of its prevalence, morbidity, impact on quality of life and cost [1–4]. Pollen [5, 6] and house-dust mites (HDM) [7] are the most common allergens related to respiratory diseases, followed by animal dander and moulds [8]. Polysensitisation, defined as the presence of more than one specific IgE sensitisation against non-related allergen sources, is frequent in Spain [6]. However, the exact clinical relevance of each sensitisation is sometimes difficult to establish and, consequently, allergists may have difficulty in deciding which is the most appropriate composition for allergen immunotherapy (AIT) for each patient. For this purpose, the recognition of the sensitisation profile (identification of primary sensitisation markers with respect to detection of specific IgE against cross-reactive allergen molecules) has been suggested as a tool to define better the relevant or irrelevant allergens in each patient [5].

The efficacy of AIT in monosensitised patients has been proven in both children and adults and with both routes of administration, subcutaneous (SCIT) and sublingual (SLIT) [9–11]. There is no agreement on the therapeutic approach to polysensitised subjects. Thus, while monoallergen AIT is preferred in European countries, the use of more than eight allergens in the same vaccine is common in the US [12]. In this regard, the European Medicine Agency (EMA) recommends that allergists restrict the mixture of non-related allergens to a minimum and advises not to mix seasonal and perennial allergens, or allergens with proteolytic activity without justification [13]. Taking these concepts together, define precisely the diagnosis seems important before prescribing AIT [14]. The present study was designed to explore the medical opinion of a panel of 62 Spanish allergists in order to achieve consensus regarding diagnosis and treatment with AIT in polysensitised patients, aiming to improve allergic treatment.

## Methods

**Figure 1:**
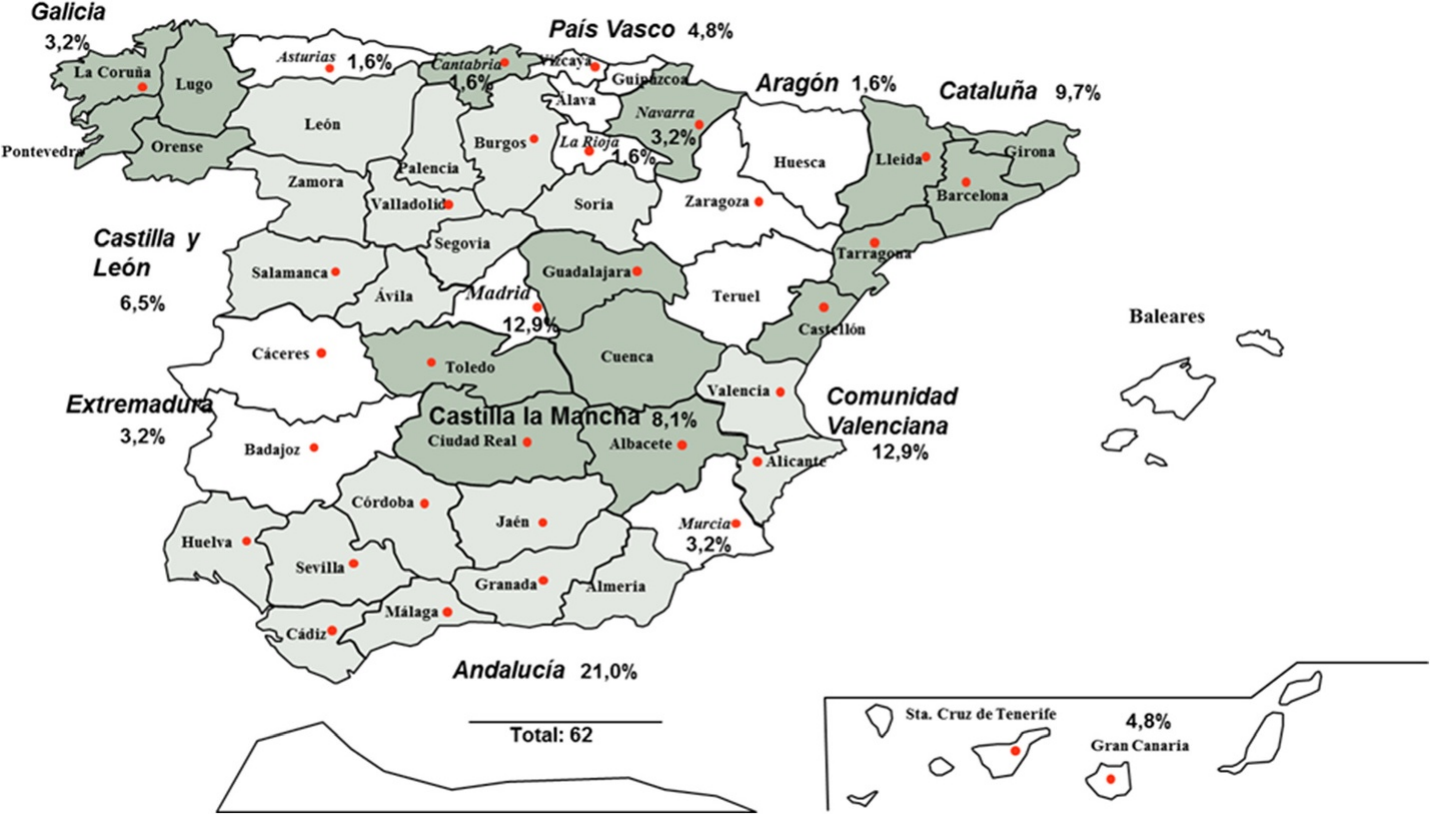
**Distribution of Spanish allergist who took part in the consensus.**

To investigate the opinion of a sample of 62 allergists from Spain (Figure 1), a modified Delphi method [15] was used. First, a steering committee of five allergists (authors of this manuscript) reviewed the medical literature on the topic and discussed the main items to be included in a structured questionnaire. Fifty-seven more allergists with a solid clinical experience in AIT in adults and/or children and a teaching clinical practice of AIT in fellows in training and in continuing medical education Spanish programmes were selected by the steering committee. This selection process began with a specific proposal from each member of the steering committee, who named 30 allergists throughout the country known for their interest in AIT to work on the project. All names repeated at least twice were included as “panelists by agreement”. The steering committee together with this team of allergists defined the final questionnaire.

### Questionnaire and Delphi methodology

The final version of the questionnaire included 88 items distributed in four topics: 1) general approach to polysensitised subjects (n = 24) (Table 1); 2) sensitisation profile regarding mite, animal dander and moulds (n = 26) (Table 2); 3) grass and olive pollen co-sensitisation (n = 18) (Table 3), and 4) other pollen polysensitisation profile (n = 20) (Table 4) representing the main geographical patterns of sensitisation in Spain. For all topics including allergens, items were grouped according to epidemiology, clinical relevance and therapeutic strategy (Tables 2A, 3A and 4A). An independent team, consisting of 3 non-medical people worked as facilitators, requesting the opinion of each allergist, using the online survey (88 items), on an individual and anonymous basis. Answers were collected from May 2013 to June 2013. After analysing the results of the first round, one of the facilitators provided an anonymous summary of the forecasts as well as the reasons allergists provided for their judgements. Thus, allergists were encouraged to revise their earlier answers in light of the replies of other members of the panel and a second round was performed dealing with those questions lacking. A nine-point, single, ordinal, Likert-type scale was used to assess the opinion on each item. Following Delphi categorization, responses were classified in three regions: (1–3) = “disagree”; (4–6) = “neither agree nor disagree”; (7–9) = “agreement”. The survey also offered the possibility of adding individual explanatory observations to every answer.

**Table 1:** **Items included in the questionnaire about “General approach to polysensitized subjects” (1A) and results (1B)**

1A)	1B)				
**Items**	**Mean**	**Median**	**% Panellists against**	**IQ**	**Result**
**Diagnostic approach to a patient with suspected respiratory allergy**					
1- Skin-tests are sufficient for the correct aetiologic diagnosis of patients with respiratory allergy	3.08	3	32.26	2	**D**
2- The size of the wheal is useful in the clinically relevant allergen identification	3.75	3	35	2.5	**NC**
3- A positive skin-test indicates the clinical relevance of the allergenic source	2.11	2	9.68	1.5	**D**
4- The specific IgE determination and quantification help us to establish the clinical relevance of an allergenic source	6.22	7	28.33	1	**A**
5- Molecular diagnosis serves to differentiate primary sensitisation from cross-reactivity	7.84	8	6.45	1	**A**
6- In molecular diagnostic tests, a cut-off that allows us to differentiate relevant allergens does not exist	6.97	7	27.42	2	**A**
7- The patient diagnostic approach must be similar, independent of whether respiratory symptoms are persistent or intermittent	6.92	8	25.81	3	**A**
8- The directed medical history and symptoms-exposure schedule allows us to identify the responsible allergenic source of the patient’s clinic in some cases	7	7	11.67	0	**A**
9- Organ-specific provocation tests are not useful in daily clinical practice due to their difficult interpretation and because they are time consuming	7.1	8	16.67	1	**A**
10a- The polysensitised patient is one who presents sensitisation to various allergenic sources	7.47	8	11.67	2	**A**
10b- The polyallergic patient is one who presents sensitisation with demonstrated clinical relevance	7.90	8	8.33	1	**A**
11- The aerobiological information should include the allergenic load in the environment	7.42	8	14.52	1	**A**
**Determinant criteria in immunotherapy prescription**					
12- Before immunotherapy prescription to a polysensitised patient, an organ-specific provocation with all suspected relevant allergens must be conducted	2.4	2	17.74	2	**D**
13- Assessment of the intensity of symptoms and medication consumption in relation to allergenic exposure should be habitual practice in immunotherapy prescription	8.23	8	1.61	1	**A**
14- Immunotherapy should only be used based on clinical studies that follow current guidelines	6.92	7.5	29.03	2.5	**A**
15-Immunotherapy prescription is advised only if relevant allergen sources are identified	7.22	7	6.67	1	**A**
**Immunotherapy composition**					
16- Enzymatic activity (proteolysis) over others should not be used	7.6	8	14.52	2	**A**
17- It is acceptable to include up to two or three allergens in one vaccine if their relevance is identified	6.28	7	28.33	2	**A**
18- Immunotherapy should include all relevant allergenic sources	3.63	3	25	1	**D**
19- Safety studies of a given extract are applicable to all extracts from identical allergenic sources	2.31	2	14.52	2	**D**
20- Efficacy studies of a given extract are assimilable to all extracts from identical allergenic sources	2.55	2	17.74	2	**D**
21- The extract mixture has a nonspecific positive therapeutic effect despite dosage reduction of included allergens	4.27	5	46.67	2	**NC**
22- If mixtures of several allergenic sources are used in immunotherapy, it is necessary to ensure the effective concentration of each one in the final composition	7.89	8	6.45	2	**A**
24- The dose–response studies are conducted with vaccines from one allergenic source so the results cannot be extrapolated to those of mixtures	7.16	8	22.58	1.5	**A**

A = Agreement; D = Disagreement; NC = No Consensus; IQ = Interquartile range.

% panellists = percentage of panellists out of the median region.

**Table 2:** **Items included in the questionnaire about “Allergy to mites, animal dander and moulds” (2A) and results (2B)**

2A)	2B)				
**Items**	**Mean**	**Median**	**% Panellists against**	**IQ**	**Result**
**Epidemiology**					
25- Knowledge of the predominant type of mites in a geographical area is useful in defining the composition of immunotherapy in an allergic patient.	7.98	8	3.23	2	**A**
26- The moulds with the most epidemiological importance in respiratory allergy are Aspergillus and Alternaria.	7.74	8	6.45	2	**A**
27- In patients with double sensitisation to mites and Alternaria, it is essential know if there is exposure to both sources	7.18	8	22.58	2	**A**
**Clinical relevance**					
28- Skin-tests, by themselves, are sufficient for the diagnosis of mite allergy, epithelia and mould.	3.57	3	25	1.5	**D**
29- Skin-tests are sufficient for the immunotherapy prescription to mites, epithelium and mould.	3.43	3	26.67	2	**D**
30- Quantitation of specific IgE in serum against full mite extract adds additional value to the skin-test.	6.4	7	26.67	1.5	**A**
31- In order to know the true sensitisation profile of patients allergic to mites, molecular diagnosis is necessary.	6.13	7	38.33	2	**NC**
32- The specific IgE determination in serum against Der p 1 and Der p 2 is useful if these components have been quantified in the vaccine.	6.83	7	20	0.5	**A**
33- The IgE specific quantification to Dermatophagoides and/or Lepidoglyphus and/or Blomia helps to decide AIT composition.	6.65	7	16.67	0	**A**
34- Alt a1 determination as primary sensitiser to Alternaria is recommended before AIT prescription with this mould.	6.18	7	35	2	**NC**
35- Clinical relevance of double sensitisation to Alternaria and mites may be improved by symptom’s calendar.	6.2	7	40	2	**NC**
**Therapeutic strategy (AIT for mite sensitised patients)**					
36-Given the high cross-reactivity between Dermatophagoides pteronyssinus and farinae, the choice of the species present in the vaccine is irrelevant.	6.45	7	26.67	2	**A**
37- Quantification of major molecular components (Der p 1 and Der p 2) in the vaccine is mandatory	6.92	8	22.58	1	**A**
38- In case of sensitisation to Lepidoglyphus or Blomia in a patient allergic to Dermatophagoides, only after proving clinical relevance of these minor mites should a vaccine be indicated.	7.47	8	14.52	1	**A**
39- Vaccines containing minor mites should not be used until efficacy has been proven.	5.97	7	40	3	**NC**
40- In case of true allergy to both a minor mite and Dermatophagoides, a vaccine containing both allergens could be used as a 50% mixture.	6.25	7	30	2	**A**
41- It is not advisable to mix mites with any other different allergenic source due to their proteolytic activity	6.37	7	28.33	3	**A**
**Therapeutic strategy (patient allergic to moulds (one or more) and with/without mite allergy)**					
42- Patients allergic to *Alternaria* only should receive a vaccine containing this allergenic source.	7.69	8	11.29	2	**A**
43- *Alternaria* vaccine must have its major allergen quantified (Alt a 1)	7.95	8	11.29	2	**A**
44- Immunotherapy with mould mixtures is not indicated.	7.15	7	23.33	1	**A**
**Therapeutic strategy (Regarding patients allergic to epithelia with/without other sensitivities)**					
45- There is not sufficient scientific evidence in immunotherapy to epithelia different from cat and dog	5.02	6	86.67	4	**NC**
46- Studies with cat epithelium vaccine have shown clinical efficacy	7.76	8	9.68	2	**A**
47- In case of mite and epithelia sensitisation, both clinically relevant when animal avoidance is not possible, a mixture of both would be advisable.	3.24	3	25.81	2	**D**
48- In case of mite and epithelia sensitisation, both clinically relevant when animal avoidance is not possible, two vaccines should be used	6.74	7.2	32.26	2	**A**
49- In the same situation as previously described, the use of a vaccine containing one allergen could be recommended followed by the consideration for a second AIT	7.37	8	19.35	2	**A**
50- In patients allergic to horse epithelium , when occupational exposure and/or severe impact on quality of life is present, AIT could be considered	8.16	8	6.45	1	**A**

A = Agreement; D = Disagreement; NC = No Consensus; IQ = Interquartile range.

% panellists = percentage of panellists out of the median region.

**Table 3:** **Items included in the questionnaire about “Olive and grass pollen allergy” (3A) and results (3B)**

3A)	3B)				
**Items**	**Mean**	**Median**	**% Panellists**	**IQ**	**Result**
**Epidemiology**					
51- All olive-grass cosensitised patients are equal if they are from the same geographic area	2.15	2	9.68	2	**D**
52- All olive-grass cosensitised patients are equal if they have the same size wheals	1.84	1	4.84	1.5	**D**
53- The genuine sensitisation components (Phl p 1–5 and Ole e 1) are suitable for identifying phenotypes	7.1	7	11.29	1	**A**
**Clinical relevance**					
54- Geographic and aerobiological data are very important in identifying the clinical relevance of olive and grass cosensitised patients	7.29	7	11.29	1	**A**
55- In olive-grass cosensitised patients, skin-tests have major limitations in confirming the clinical relevance	7.18	7.5	16.13	1	**A**
56- A higher level of olive IgE than grass (or vice versa) is useful to highlight the clinical relevance in cosensitised patients	4.08	3	45	2	**NC**
57- Confirmation of true sensitisation to olive or grass requires an IgE against genuine components	7.56	8	8.06	1	**A**
58- The clinical relevance in olive-grass cosensitised patients can only be defined by organ-specific provocation tests	3.19	3	25.81	2	**D**
**Therapeutic strategy (Immunotherapy indication)**					
59- The identification of the relevant allergenic source in patients cosensitised to grass and olive is essential before prescribing immunotherapy	7.98	8	4.84	1	**A**
60- The demonstrated efficacy in grass immunotherapy is the same as 50% mixture with olive	2.92	3	20	1	**D**
61- In the case of olive-grass cosensitised patients, it is preferable to formulate personalised mixes (variable percentages of the two extracts)	4.52	5	71.67	4	**NC**
62- As grass and olive are complex extracts, the dosage of individual allergens in immunotherapy would be appropriate	7.05	7	10	1	**A**
63- The olive-grass cosensitised patient should not receive immunotherapy until more efficacy data is obtained	2.55	2	14.52	2	**D**
**Therapeutic strategy (Immunotherapy formulation)**					
64- The lack of knowledge of therapeutic doses makes the formulation of mixtures difficult	6.82	7	27.42	2	**A**
65- It is correct to formulate olive-grass personalised mixes depending on the size of the wheal	2.53	2	22.58	2	**D**
66- It is preferable to formulate olive-grass personalised mixes (varying percentages of the two extracts) in proportion to the IgE amount corresponding to the two extracts	3.72	3	46.67	3.5	**NC**
67- If the manufacturer guarantees the individual doses of allergens, mixtures of different Poaceae and olive varieties are irrelevant	6.95	7	23.33	1	**A**
68- Grass and olive personalised mixtures (varying percentages of the two extracts) should never be used	3.92	3	30	2	**D**

A = Agreement; D = Disagreement; NC = No Consensus; IQ = Interquartile range.

% panellists = percentage of panellists out of the median region.

**Table 4:** **Items included in the questionnaire about “Allergy to other pollens” (4A) and results (4B)**

4A)	4B)				
**Items**	**Mean**	**Median**	**% Panellists**	**IQ**	**Result**
**Epidemiology**					
69- Pollen respiratory allergy does not always have a seasonal character	7.79	8	8.06	2	**A**
70- In the allergologic diagnosis, it is essential to know the allergenic sources and preferential exposure calendars from the geographic area	8.37	8.5	0	1	**A**
71- In the allergologic diagnosis, it is essential to know the sensitisation prevalence to different allergenic molecules from the geographical area	7.77	8	6.45	2	**A**
72- It is essential to know the aerobiology area: more captures are needed	7.81	8	8.06	2	**A**
**Clinical relevance**					
73- The diagnosis of allergy to *Parietaria* can sometimes be hampered by the co-sensitisation to other allergen sources, such as dust mites	6.84	7	25.81	2	**A**
74- Sensitisation to *Parietaria* pollen represents a major challenge for the establishment of clinical relevance, because pollination coincides with other relevant allergenic sources	7.35	7.5	12.9	1	**A**
75- Clinical data (symptoms, time of symptoms, their duration) and aerobiological data are the most important tools for the diagnosis of primary sensitisation sources in patients sensitised to multiple pollens	6.85	7	20	1	**A**
76- In pollinic polysensitised patients, panallergen skin-tests (profilin, LTP and polcalcin) are useful for selecting relevant allergens	6.82	8	25.81	2	**A**
77- The molecular diagnosis does not provide more information than skin-tests, as most sensitisation is due to relevant molecular allergens rather than panallergens	4.85	3	48.33	4	**NC**
78- In a polysensitised patient, the presence of symptoms during the pollinic period of given pollen does not imply clinical relevance of this allergenic source	5.22	5.5	88.33	4	**NC**
79- Molecular diagnostics is limited by the supply of molecular constituents of low prevalence pollens	6.87	7	11.67	1	**A**
**Therapeutic strategy**					
80- Immunotherapy is contraindicated in polysensitised patients with more than two clinically relevant pollen types	2.66	2.5	17.74	2	**D**
81- Patients polysensitised to pollens with polcalcin sensitisation do not benefit from treatment with immunotherapy because they have a higher number of reactions	4.1	5	45	2	**NC**
82- The administration of more than one vaccine could be indicated for patients sensitised to more than one relevant allergenic source	6.63	7	20	0	**A**
83- Immunotherapy prescription with different allergenic sources is only justified in the case that pollination periods from such sources do not coincide	2.92	3	22.58	1	**D**
84- When prescribing immunotherapy with a mixture of several allergenic sources, only those pollens with significant exposure should be considered	7.07	7	11.67	0	**A**
85- In areas where grass and olive are prevalent allergens, if profilin is positive, only grass must be included in the vaccine, although it is also common to find sensitisation to other pollens as well	4.03	3	35	3.5	**NC**
86- If mixtures of several allergenic sources are used in immunotherapy, they should include a higher percentage, depending on the clinical relevance of each geographical area	4.58	5	66.67	4	**NC**
87- Immunotherapy would only be indicated if quality extracts for these pollens exist	7.81	8	6.45	2	**A**
88- The efficacy of immunotherapy is associated with an early indication	7.31	7.5	19.35	1	**A**

A = Agreement; D = Disagreement; NC = No Consensus; IQ = Interquartile range.

% panellists = percentage of panellists out of the median region.

**Figure 2:**
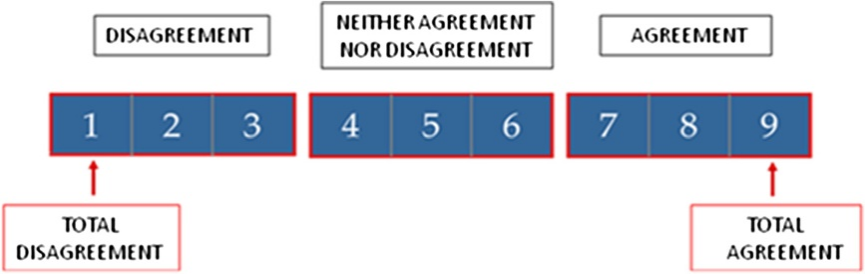
**Valuation scale of professional criteria or clinical recommendations proposed to be judged.** 1–3: I disagree with the assertion (a lower score indicates greater level of disagreement); 4–6: Neither agree nor disagree with the assertion; I do not have fully defined criteria about the question (choose 4 or 6 if closer to disagreement or to agreement, respectively); 7–9: I agree with the assertion (a greater score indicates greater level of agreement).

Once the second round was finished, the results were analysed. The median position of scores and the “level of agreement or disagreement” [16] achieved was measured according to the following criteria: an item is considered to have consensus when no more than a third of the scores are found outside of the region of three points (1–3),(4–6),(7–9) where the median is located. In this case, the value of the median score determines the group consensus reached: “agreement” majority with medians ≥7; “disagreement” majority with medians ≤3; “no consensus” items with medians in the region 4–6 and when the scores of a third or more of the participants are in the region 1–3, and another third or more in the region 7–9. Also considered for reassessment were the items where a high dispersion of opinions (interquartile-range ≥ 4points) (Figure 2) was observed.

## Results

Detailed results of each item (mean, median, percentage of distribution of respondents located outside the region of the median, interquartile range and consensus result) are depicted in Tables 1B, 2B, 3B and 4B. Overall, consensus was reached in 73/88 items (83%), 55 of them (63%) were in terms of agreement and the remaining 18 items (20%) in terms of disagreement with the assertion presented.

### General approach to polysensitized subjects

Consensus was reached on 22/24 items, 16 in terms of agreement and six items in terms of disagreement. Two items were left without consensus. Specifically, when considering the size of the wheal obtained in SPT, some doctors supported its usefulness to define the clinical relevance of an allergen while others did not. Consequently, no consensus was achieved for this specific statement (item number 2, Table 1B). Even though participants gave more value to serum specific IgE determinations than to SPT for identifying clinically relevant allergens, they seemed to rely more on molecular diagnosis but the lack of an exact cut-off level of serum specific IgE against molecular compounds that could assist this purpose is still not established. As regards to AIT, the prescription should be based on scientific evidence and only extracts with proven efficacy should be used. Vaccines containing up to two to three allergen sources could be used if more than one allergen appears to be relevant but no consensus was found when deciding if a mixture has or not a nonspecific positive therapeutic effect.

### Sensitisation profile involving mite, animal dander and moulds

Consensus was reached on 21/26 items, 18 with overall agreement and three in terms of disagreement. Consensus was not reached on the remaining five items (Table 2B). Thus, even though allergists think that serum specific IgE against groups Der 1 and Der 2 could be helpful if these allergens are quantified in the allergen extract, no consensus was achieved regarding the value of these molecular compounds to better identify the sensitisation profile of patients allergic to mites (item number 31). Minor mites such as *Lepidoglyphus* and *Blomia* represent a diagnostic problem because of the lack of clinical trials using AIT for them and consensus was not reached on this point. Nevertheless, in circumstances where sensitisation to *Dermatophagoides* and minor mites coexists and clinical relevance for both types of mites is suspected, a vaccine containing both species of mites could be suitable.

Participants also gave their opinion on mould and animal dander allergy in this section. As far as mould allergy is concerned, Quantification of Alt a 1 in the vaccine is highly recommended, even after not reaching consensus with respect to the need for quantifying serum specific IgE against this main allergen of *Alternaria* in sensitised patients. In case of double sensitisation to mites and *Alternaria*, no consensus was reached regarding the value of the evaluation of symptoms after exposure to both. Finally, in relation to animal dander sensitisation, AIT with cat and dog epithelia has demonstrated its efficacy and both may be used but no consensus was achieved in case of animals different from cat and dog.

### Grass and olive pollen co-sensitisation

Consensus was achieved in 15/18 items, eight of them in terms of agreement and seven in terms of disagreement with the assertion mentioned (Table 3B). Results obtained in this section of the questionnaire marked the limitations of SPT and serum specific IgE against grass and olive pollens as complete allergenic sources for clearly establishing the allergenic profile of these patients. However, performing specific IgE against specific allergens such as Phl p 1, Phl p 5 and Ole e 1, considered as markers of primary sensitisation, helps to discriminate between a genuine sensitisation pattern and cross-reactivity without clinical relevance. Selection of the vaccine should be made depending on such results, and only when double genuine sensitisation assessed by molecular diagnosis was confirmed should vaccination with both allergens be performed.

The use of personalised formulas with different percentages of olive and grass extracts in the same vaccine, based on size of the wheal or the value of specific IgE, is still a matter of debate and no consensus was reached. In fact, some panellists are still in favour of varying the proportion of allergens for individual patients.

### Other pollen (weeds and trees) polysensitisation profile

**Figure 3:**
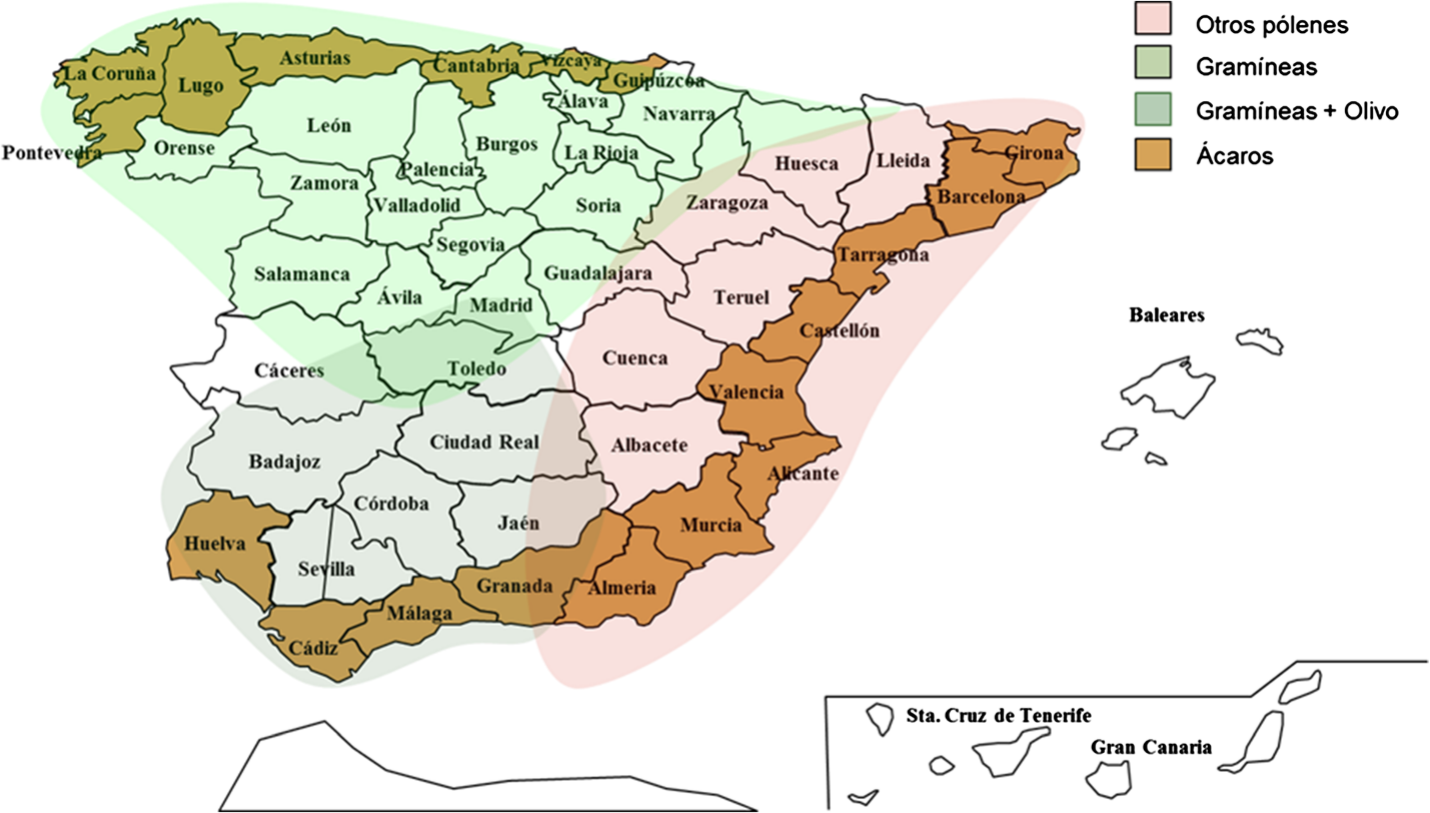
**Main geographical areas based on patients’ sensitisation profile.**

Participants are aware of clinical problems in identifying relevant allergens in areas with high and varied pollen exposure and overlapping pollination periods. For that reason, only 15 out of 20 items agreed, 13 in a positive way and 2 in a negative one (Table 4B). The lack of a clearly established seasonal pattern for some pollen hinders the diagnosis. And, in this case, molecular diagnosis is of little help because of the low availability of molecular compounds of pollens apart from grass and olive. Interpretation of polcalcin sensitisation is not always possible and could act as a confounding factor. For that reason, a deep knowledge of all allergenic sources from each zone (Figure 3), including pollen calendars and specific taxons is essential. No consensus was achieved regarding the utility of the sIgE to panallergens (profilin or polcalcin) in complex pollen areas because of difficulties in recognising the relevant and primary sensitiser. The same occurred when profilin or polcalcin were assessed as relevant grass sensitisation markers that would allow excluding other allergens in the AIT. In the case of mixing several allergens, a consensus was reached regarding the use of those pollens with demonstrated clinical relevance, notable exposure, no more than 3 allergens in the same vaccine, and always with quality extracts. Defining the composition of the mixtures is an ongoing problem without consensus. An accepted alternative is the possibility to administer ≥2 vaccines in some cases (although in practice it is assumed that the administration of 2 vaccines is not always acceptable due to the economic cost).

## Discussion

### General approach to polysensitised subjects

Polysensitisation is a serious problem in countries like Spain, where different allergenic sources may influence clinical symptoms in allergic patients. Allergists from Spain are used to dealing with this problem but, on some occasions, lack of evidence could make them hesitate about the best treatment option in each case. Other Mediterranean countries such as Italy or Greece share the same problem [17, 18]. This lack of scientific evidence led us to promote the present Consensus Report aiming at helping allergists with diagnosis and treatment in complex areas from an allergological point of view. As authors, we are aware of the limitations of expert opinion statements and for this reason the well-known and highly contrasted Delphi Method was used to gather and analyse the proposed items reflecting common problems in clinical practice. As a whole, 83% of the items agreed, but consensus was not similarly reached if we consider each group of items separately. It seems that decisions are easier when considering general situations (91.6% agreement) than when deciding specific solutions for specific allergens. It seems that experts have the skills to decide how to act but they fail to agree when diagnostic tools are limited or are not available. According to this, the lowest level of agreement, 75%, was reached in the “other pollen sensitisation profile”, reflecting difficulties in recognising relevant allergens in polysensitised patients in those areas, such as Mediterranean Spanish coast, with a high number of allergens and high allergen pressure [19].

According to what has been published, experts agree with respect to the limited value of SPT in firmly recognising relevant allergens [20]. SPT is commonly used in daily clinical practice because of its simplicity and reliability to quickly identify IgE sensitisation to inhalant allergens. The relationship between SPT wheal sizes and the clinical relevance of positive results is debatable [21, 22] and, consequently, it is understandable that consensus was not reached on this point. In fact, many factors can influence SPT results. A seasonal variation in wheal sizes has been described for several allergens [23]. The site where SPTs are performed may influence the results. The ulnar side of the arm is more reactive than the radial side, and the antecubital fossa is more reactive than the wrist [24]. Finally, the SPT technique may influence the size of the wheal reaction [22]. Experts consider serum specific IgE more reliable in recognising clinically relevant allergens, as it has been considered by others [25]. Recent advances in molecular diagnosis are supposed to help allergists in the recognition of genuine sensitisations, and experts agree on the value of this diagnostic tool, but the lack of a cut-off level is a limitation of the test. The fact that sensitisations are not always accompanied by clinical symptoms is well-recognised [26]. For that reason, experts agree that polysensitisation is not the same as polyallergy, meaning that polyallergic patients are only those with clinically relevant symptoms related to allergen exposure. To identify relevant allergens, symptom scores are useful in a similar way than when performing clinical trials. The recognition of a cause-effect relationship should be done according to the allergen exposure. Aerobiological information is essential for this purpose. Even though allergen challenge tests can be useful in clinical trials and even in proving the clinical relevance of purified allergens (profilin, polcalcin and so on) [27], they are time-consuming, and therefore the implementation of these tests in daily clinical practice before prescribing AIT is not necessary. The use of allergenic extracts with proven efficacy is strongly supported and clinical studies should follow the guidelines of regulatory agencies [13]. Taking into account differences among products from different allergen manufacturers, each one must perform their own clinical trials for every single product, since results from one study with one allergen cannot be extrapolated to a different allergen, or even the same allergen produced by another manufacturer [28–30]. Finally, when considering mixing allergens and following EMA suggestions, non-related allergens with possible enzymatic activity should not be mixed; even for related allergens, no more than three should be used in the vaccine.

### Sensitisation profile involving mite, animal dander and moulds

According to what has been accepted in the general approach section, experts agree with the idea of knowing the species of mites predominant in one area. It is well-known that mites are not uniformly distributed all over the world and, even for the same *Dermatophagoides* genus, distributions of *pteronyssinus* and *farinae* species vary from place to place [7, 31]. However, the high degree of cross-reactivity proven between them [7, 32] seems not to be a problem when selecting the composition of AIT. That is not the case for minor mites. The lack of cross-reactivity among them and *Dermatophagoides*[33–35] represents a problem difficult to resolve. Thus, while experts could recommend the prescription of a vaccine containing minor mites and *Dermatophagoides* in equal proportions, they recognise the need for more studies showing clinical efficacy of AIT with minor mites alone before strongly supporting their use as a therapeutic option. Consequently, no agreement was achieved on this specific item.

Molecular diagnosis, as far as mite and mould allergy is concerned, seems to be of little or no value, and therefore experts show no agreement regarding the need for measuring sIgE before prescribing AIT with them. In fact, no consensus was achieved when specifically asked about the need of measuring sIgE against Der p 1, Der p 2 or Alt a 1. However, quantification of major molecular compounds of both mites and moulds in the vaccine is evaluated positively by participants. One possible explanation for these contradictory results may be related to the recent advances in the recognition of the pattern of sensitisation against Der p 1 and Der p 2 and the lack of correlation between them [7]. More studies are needed to balance the importance of both molecules in each patient, but in the meantime quantification of major allergen compounds in the vaccine could be useful to explain adverse effects or even efficacy.

Biological standardisation of moulds is difficult [36] but participants consider *Alternaria* and *Aspergillus* spp the only relevant allergens from a respiratory point of view. Moreover, only *Alternaria* could be considered as a potential allergen for AIT. For that reason mixture of moulds should never be an option accepted by experts. The main problem involving *Alternaria* is co-sensitisation with mites. There is no consensus regarding the utility of symptom and medication scores or the recognition of sources of exposure when dealing with patients sensitised to mites and *Alternaria*. If needed, more than one vaccine could be used and this recommendation applies not only for mites and *Alternaria* but also for animal dander allergy. Vaccines with cat or dog allergens may be used if these allergens are clinically relevant and avoidance is not an option. AIT with horse extract is not supported by all experts, and no consensus was reached despite proven efficacy. The main limitation could be that there was only one published study when the questionnaire was done, and it is not a double-blind placebo-controlled study [37]. Recently, Nanda and Wasam have published their experience with a horse extract at different doses in eight patients, with positive results [38].

### Grass and olive pollen cosensitisation

As far as grass and olive pollen is concerned, participants agree that measurement of specific IgE to Phl p1, Phl p 5 and Ole e 1, molecular compounds considered as phenotypic markers of genuine grass and olive pollen sensitisation, respectively are useful in deciding the composition of AIT. In addition, some allergens considered minor because of their lower prevalence in terms of sensitisation, such as Ole e 7 and Ole e 9, could be of importance in regions with high allergenic pressure to olive pollen. Moreover, the presence of high levels of specific IgE against them could explain some serious adverse reactions during AIT with olive pollen extracts [38]. AIT is claimed to be a first line therapy option in patients sensitised to grass and olive, if both are clinically relevant. The low cross reactivity between pollens of different species, including oleaceae and grass [39, 40], but particularly the presence of genuine sensitizers [41, 42], justifies the consensus on the suitability of individual dosages of each extract. Finally, no consensus was achieved when experts were asked about the use of personalised immunotherapy. The term “personalised” was used to express formulations with different proportions of each allergen as opposed to the idea of fixed combinations in equal proportions (50%-50%), but the statement recorded was not clearly defined and could have been misunderstood.

### Other pollen (weeds and trees) polysensitisation profiles

Pollens from trees and weeds such as *Betula spp, Platanus spp, Cupressus spp, Artemisia spp, Chenopodium spp, Parietaria spp, Plantago lanceolata* and *Salsola kali* may induce IgE sensitisation in atopic subjects in exposed individuals in Spain and other Mediterranean countries [43]. Exposure to these pollens varies from place to place. This is why allergists from different regions may have different opinions about how to deal with polysensitised subjects, depending on the different taxons present in a particular area. In fact, the level of consensus was lower in this section compared with the others. It is noteworthy that experts do not have a recommendation regarding the usefulness of molecular diagnosis because of difficulties in distinguishing primary sensitisation and cross-reactivities. Even symptom and medication scores are of little value. Polcalcin has been considered a confusion factor, but the actual meaning of polcalcin sensitisation is far from known. There are no published articles investigating the prognostic value of such sensitisation or the possible interference on efficacy or safety of immunotherapy. As regards profilin, some authors have demonstrated a relationship between profilin sensitisation and symptoms [19] while others consider profilin sensitisation a confounding factor that could interfere with immunotherapy efficacy, because of its role as a confounding factor for the diagnosis [6]. More studies are needed to clarify these questions. In the meanwhile, patients allergic to more than one non-related pollen could be treated with more than one high-quality vaccine or a mixture of pollens with clinically relevant exposure.

## Conclusion

In conclusion, choosing the best allergen for AIT in polysensitized patients is a difficult task. Consensus like the one presented here could be of help to allergists before making decisions on AIT composition. From the results of this survey we can conclude that:

- SPTs are not enough to accurately diagnose allergy in polysensitised patients.
- An approach to molecular diagnosis seems to be useful in pollen-allergic patients if all relevant molecules were available.
- AIT prescription should be based on scientific evidence, and only be indicated if relevant allergens are identified.
- Data from clinical records and knowledge of allergen exposure in each area are essential to define AIT composition.
- No more than three allergenic sources should be mixed in the same vaccine.
- Each vaccine should have its own safety and efficacy studies.
